# Environmental Controls on River Assemblages at the Regional Scale: An Application of the Elements of Metacommunity Structure Framework

**DOI:** 10.1371/journal.pone.0135450

**Published:** 2015-08-13

**Authors:** Jonathan D. Tonkin, Andrea Sundermann, Sonja C. Jähnig, Peter Haase

**Affiliations:** 1 Department of River Ecology and Conservation, Senckenberg Research Institute and Natural History Museum Frankfurt, D-63571 Gelnhausen, Germany; 2 Leibniz-Institute of Freshwater Ecology and Inland Fisheries (IGB), Department of Ecosystem Research, 12587 Berlin, Germany; University of Waikato (National Institute of Water and Atmospheric Research), NEW ZEALAND

## Abstract

Understanding factors that structure regional biodiversity is important for linking ecological and biogeographic processes. Our objective was to explore regional patterns in riverine benthic invertebrate assemblages in relation to their broad positioning along the river network and examine differences in composition, biodiversity (alpha and beta diversity), and environmental drivers. We up-scaled methods used to examine patterns in metacommunity structure (Elements of Metacommunity Structure framework) to examine faunal distribution patterns at the regional extent for 168 low-mountain stream invertebrate assemblages in central Germany. We then identified the most influential environmental factors using boosted regression trees. Faunal composition patterns were compartmentalised (Clementsian or quasi-Clementsian), with little difference from headwaters to large rivers, potentially reflecting the regional scale of the study, by crossing major catchment boundaries and incorporating different species pools. While idealised structures did not vary, environmental drivers of composition varied considerably between river sections and with alpha diversity. Prediction was substantially weaker, and the importance of space was greater, in large rivers compared to other sections suggesting a weakening in species sorting downstream. Further, there was a stronger transition in composition than for alpha diversity downstream. The stronger links with regional faunal composition than with richness further emphasises the importance of considering the alternative ways in which anthropogenic stressors are operating to affect biodiversity patterns. Our approach allowed bridging the gap between local (or metacommunity) and regional scales, providing key insights into drivers of regional biodiversity patterns.

## Introduction

The metacommunity concept set the scene for a new wave of spatial ecology, where the notion of dispersal linking spatially separated communities and thus the interplay between local (niche) and regional (dispersal) influences is central [1,2]. Therefore, a major focus of research in recent years has been disentangling the relative influence of environmental vs. spatial processes [3–5], and particularly the interplay between species sorting (SS) and mass effects (ME). Consensus has indicated that SS is more commonly the prevailing paradigm of the four originally outlined paradigms, followed by ME (also including neutral model and patch dynamics) [6]. Both of these paradigms arise from traditional niche theory, where species have a preferred niche [2]. On the one hand, SS reflects adequate dispersal to allow strong environmental structuring of communities. On the other hand, ME results from dispersal ‘flooding’ strong niche control through a net flow of individuals into non-preferred habitats and thus a weaker local environmental compared to spatial signal emerges [2].

Given this central role of dispersal, the structure of the dispersal network should be a key component shaping metacommunities, such as the difference between simple lattice grids and dendritic networks [7]. Riverine networks are inherently dendritic, which promotes unique metacommunity dynamics [7,8], and dispersal mode within these networks can strongly influence metacommunity structure of aquatic organisms [9]. Given their dendritic structure, dominant structuring mechanisms may shift from SS in headwater streams to ME in sections immediately downstream [10], but patterns over larger gradients in stream size or at larger spatial scales are less certain. This should arise through the higher level of connectivity in downstream sections resulting in dispersal swamping environmental or niche control [10]. Consequently, headwaters can support high levels of biodiversity, particularly at the beta diversity level [11,12].

Freshwater systems are under significant threat from anthropogenic stressors [13,14]. However, while the influence of anthropogenic stressors on alpha diversity in freshwater systems is relatively well understood, how they influence metacommunities is much less clear [15]. There are fundamental differences in typical commonly applied diversity measures (e.g. number of taxa, regardless of taxonomic identity) and measures of community and metacommunity structure (e.g. changes in composition among sites, incorporating taxonomic identity and/or abundances). Therefore, it is important to disentangle how these abiotic controls shape river metacommunities and regional assemblage patterns, with recent studies having begun to disentangle some of the more indirect influences of anthropogenic stress on riverine communities, including beta diversity components and co-occurrence patterns [16,17]. Understanding the abiotic factors best associated with community composition or metacommunity structure should benefit the protection of biodiversity, particularly if these factors differ from those shaping local species richness.

An alternative way to examine metacommunities is to determine which idealised structure an assemblage fits best through the Elements of Metacommunity Structure (EMS) framework [18,19]. In addition to the ability to compare the structure with environmental gradients, one of the benefits of EMS is that it can be extrapolated to test patterns at regional scales beyond the spatial scale of typical metacommunities for which it was originally intended [20–22]. This is important as, at increasing spatial scales, the lines begin to blur between the disciplines of metacommunity ecology, biogeography and macroecology [23].

The EMS approach allows for comparing the empirical structure of assemblage patterns with several idealised distributions. For instance, checkerboard distributions result where species distributions are arranged in a mutually exclusive manner, originally considered to emerge through intense competition between species [24], but can also emerge through other factors, such as species’ environmental preferences or historical influences [25]. Many other distributions are possible. For example, where species respond to an environmental gradient in clumped groups (i.e. Clementsian) [26] or where responses of species are more individualistic (i.e. Gleasonian) [27]. Alternatively, assemblage patterns can be arranged as nested subsets, where species-poor communities are nested within other communities, often resulting from the characteristics of species [28,29].

Our central objective was to explore regional patterns in riverine benthic invertebrate assemblages in relation to their broad positioning along the river network and examine differences in composition, biodiversity (alpha and beta diversity), and environmental drivers. To do this, we up-scaled the EMS approach [18,19] to assess regional faunal patterns (beyond the scale of a strict metacommunity). We related these gradients in faunal composition to environmental variables and compared patterns between headwaters, mid-sized streams and large rivers. We also assessed whether factors influencing faunal composition patterns differed between those driving richness patterns. As we examined patterns at spatial scales bordering metacommunity ecology and biogeography, we could not accurately examine hypotheses based on previous stream metacommunity studies. However, an interesting question in stream metacommunity ecology in recent years, emerging from general metacommunity theory, is whether or not there is a transition from SS to ME from headwaters to mainstems through changes in the level of connectivity of sites [10]. Therefore, we were interested in whether compositional patterns and environmental control differed between different sections along the river network at scales beyond those typically addressing this question.

We asked the following questions: (1) Which idealised structure, from the EMS framework, best fits the empirical data, and does this differ between headwaters, mid-sized and large rivers? (2) Is there a stronger predictive ability of environmental variables in headwater streams compared to sites further downstream, reflecting differences in the relative control of environmental and spatial influences (i.e. SS or ME)? (3) If the level of environmental control does differ between headwaters, mid-sized and large rivers, does it lead to the pattern of alpha diversity increasing progressively downstream, but beta diversity declining [12]? (4) Does the importance of chemical variables increase from headwaters to large rivers, due to higher levels of catchment stressors present in downstream sections? (5) Do drivers of faunal composition largely match those shaping local species richness?

## Materials and Methods

### Study sites

**Fig 1:**
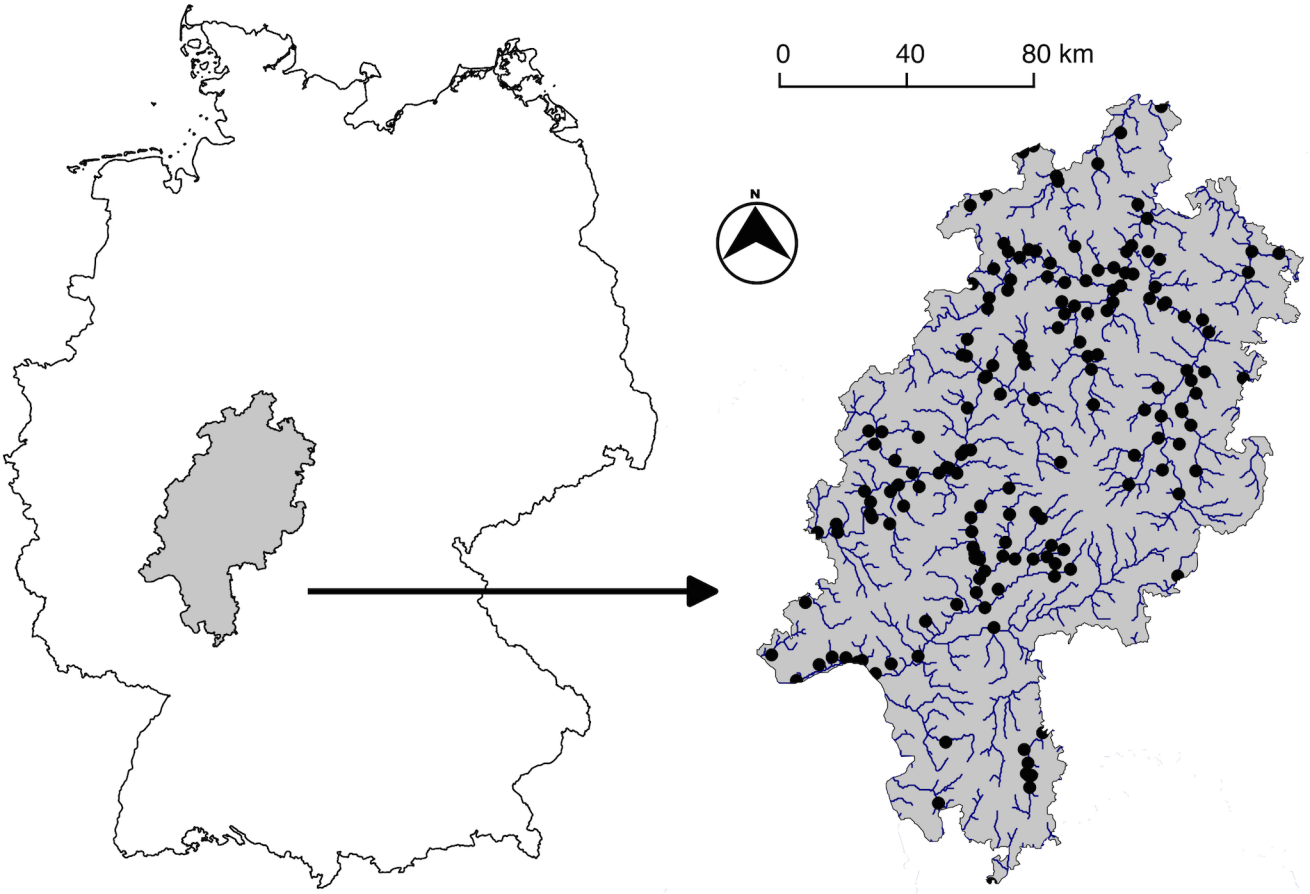
Map of study sites. Map of 168 low-mountain streams and rivers in the German state of Hessen, sampled between 2005 and 2008.

We compiled a dataset of benthic invertebrates, physical properties, catchment land use and in-stream chemical variables from 168 low mountain streams (catchment sizes: 3–975 km^2^) sampled in the German state of Hesse in central Germany, collected between 2005 and 2008. Each site was only sampled once for our study (Fig 1). This region is divided approximately across the middle by two large river drainages: the Weser River flows to the North and the Main River to the South (Fig 1).

To examine our question that faunal composition differs between sites at broadly different positions along the river network, we used catchment size as a guide to break sites into different river sections (i.e. small catchment size represents headwater streams and large catchment size represent further down the river network). Specifically, we sub-divided the data into three size-based categories using catchment size as a guide, as well as keeping the full dataset for analyses. These three categories were: headwaters (N = 48)—catchment size < 20km^2^; mid-sized streams (N = 69)—catchment size 20–100 km^2^; and large rivers (N = 51)—catchment size 100–1000 km^2^.

### Environmental data

We used a suite of environmental variables to predict richness and regional faunal composition, including catchment land use, physical properties and water chemistry (Table 1). Catchment land use was calculated for the entire upstream catchment area of each site using CORINE Land Cover database data [30], and was grouped into seven classes (Table 1).

**Table 1:** Environmental variables used to predict benthic invertebrate richness and faunal composition from 168 low-mountain streams in central Germany.

Category	Variable	Explanation/Units	Mean (S.D.)	Range
Physical	Catch_size	Catchment size in km^2^	102.11 (152.91)	3.34–975.23
	Elevation	Elevation in m asl	191.97 (60.25)	87.38–378.28
Land use	Agriculture	% of catchment agriculture	53.06 (16.61)	1.66–89.80
	Artificial	% of catchment artificial surfaces	6.18 (6.57)	0.00–47.9
	Forest	% of catchment forest	40.16 (16.96)	0.00–98.34
	Natural_bare	% of catchment naturally bare land	0.01 (0.06)	0.00–0.51
	Shrub	% of catchment shrub	0.55 (1.10)	0.00–8.93
	Water	% of catchment water	0.04 (0.20)	0.00–1.66
	Wetlands	% of catchment wetlands	0.00 (0.01)	0.00–0.07
Chemical	Ammonium	Mean ammonium (mg L^-1^)	0.18 (0.35)	0.03–2.85
	Chloride	Mean chloride (mg L^-1^)	67.86 (198.93)	8.57–1942.86
	Max_pH	Mean maximum pH per year	8.16 (0.23)	7.41–8.66
	Max_Temp	Mean maximum temperature per year (°C)	18.36 (1.6)	14.40–24.90
	Min_DO	Mean minimum dissolved oxygen (DO) per year (mg L^-1^)	8.18 (0.84)	4.95–9.67
	Min_pH	Mean minimum pH per year	7.44 (0.32)	6.35–8.06
	OP	Mean orthophosphate (mg L^-1^)	0.16 (0.14)	0.01–0.98
	TOC	Mean total organic carbon (mg L^-1^)	4.10 (1.10)	1.10–9.10
	TP	Mean total phosphorus (mg L^-1^)	0.21 (0.14)	0.02–1.01
Spatial	PCo1	Principal Coordinate 1	**-**	**-**
	PCo2	Principal Coordinate 2	-	-

Mean, standard deviation (S.D.) and range (min-max) are shown for all variables.

Data on the following physicochemical variables were available for all sites: ammonium, chloride, orthophosphate, oxygen, pH, total phosphorus, water temperature and total organic carbon. Our objective for these variables was to characterise the physicochemical characteristics of the streams in the study over time, rather than as a snapshot value. Therefore, we used variables that were collected usually monthly and over several years, between 2007 and 2014, with a mean number of all samples per site of 52.8 ± 16.3 (1 S.D), and a minimum of eight samples. Although, this does not align directly with the sampling dates, it allows a characterization of the physicochemical conditions of the study streams. These values were averaged for each year, and where a minimum or maximum value is used, it represents the mean of the annual minimum or maximum value over several years (Table 1).

We used principal coordinate analysis (PCoA) to generate vectors from a matrix of geographic distances between sites. We ran the PCoA using the vegan R package [31], and selected the first two vectors for analysis.

### Benthic macroinvertebrates

Benthic macroinvertebrate data was compiled from routine surface water surveys performed by German governmental environmental agencies between 2005 and 2008, with each stream only being included once. Sampling was performed using the official EU Water Framework Directive (WFD) compliant sampling protocols for German streams [32]. This method uses a multihabitat sampling approach according to the coverage of available microhabitats at a site. All microhabitats in a 100-m long reach were first recorded in 5% coverage units, and each sampling unit (25 x 25 cm) sampled with a 0.5-mm mesh kick net. Twenty sample units were taken from each site and then pooled for later analysis (1.25 m^2^ total sampling area). Taxa were then sorted and identified in the laboratory to consistent levels between sites according to the WFD-compliant "Operational Taxalist for Running Waters in Germany" [33]; (http://www.fliessgewaesserbewertung.de/en/download/bestimmung/).

### Statistical analysis

#### Diversity measures

All statistical analyses were carried out in R 3.0.2 [34]. Taxonomic richness was calculated as the total number of taxa present at each site. We also calculated Simpson’s diversity index [35] and rarefied taxonomic richness (adjusted for 100 individuals) [36] using the ‘diversity’ function in the vegan package [31]. As an estimate of beta diversity (variation among communities) of the different catchment categories, we used tests of the homogeneity of dispersion (PERMDISP2 [37] with the ‘betadisper’ function in vegan, based on Bray-Curtis distances, and calculated as the distance to group centroid. We assessed differences in diversity metrics between the three river sections using one-way analysis of variance (ANOVA; ‘aov’ function in R), followed up with Tukey’s HSD tests (‘TukeyHSD’ function in R) for pairwise comparisons.

#### Faunal distribution patterns

We applied the Elements of Metacommunity Structure (EMS) approach of Leibold & Mickelson [18] and Presley et al. [19] to assess patterns of faunal composition. This approach enables analysis of coherence, species turnover and boundary clumping of species distributions to explain which idealised pattern [18] or quasi-structure [19] are best associated with the observed pattern. The original EMS framework proposed by Leibold & Mikkelson [18] identified six different idealised metacommunity structures to differentiate between: random, checkerboard, nested subsets, evenly spaced, Gleasonian and Clementsian. However this has since been further developed by Presley et al. [19] to include up to 14 different structures, including quasi structures. Detailed explanation and diagrams of these structures can be found in several sources (e.g. [18,19,38]).

Analysis of EMS essentially follows a three-step approach. Firstly, coherence in distribution along a latent environmental gradient is assessed, followed by analysis in species turnover in space, followed by analysis of boundary clumping. EMS uses reciprocal averaging (RA), an unconstrained ordination method, to arrange points within a matrix in the best possible manner by maximising correspondence. That is, sites and species are arranged so that similar assemblages and distributions are adjacent, respectively. This approach has the advantage that sites are arranged based on similarity of species-specific distributions and thus allows comparison with latent environmental variables with no prior knowledge of the factors driving species distributions [18].

Ordination axes (in this case, the first two) can then be extracted to link with environmental data. The primary axis represents the best arrangement of sites with the strongest link between composition of communities and the distribution of species in space. Environmental variables that are strongly associated with the primary RA axis should therefore be important shapers of assemblage composition. While Leibold & Mikkelson [18] originally proposed using only the first ordination axis, Presley et al. [39] suggested that it can also be worthwhile examining the second axis given it can also represent important structuring components. Therefore, we also examined the second RA axis, but the second axis is constrained to be orthogonal (uncorrelated) to the first [40], and thus may represent a less clear arrangement than the primary axis.

We performed these methods in the package Metacom [41]. We created site-by-species incidence matrices (i.e. presence-absence) to assess faunal composition for all sites combined, as well as for headwaters, mid-sized streams, and large rivers. We excluded any taxa that were found at less than two sites as these can bias the EMS results, particularly coherence and boundary clumping patterns [39]. We chose *a priori* to use a null model that constrained simulated species occurrences (i.e. column totals) to be proportional to their incidences within the matrix and simulated row totals (site richness) to equal the empirical richness (method "r1" or fixed-incidence proportional; [42]). We generated 1000 random matrices with the above distribution constraints to compare our empirical matrix against.

Firstly, coherence in community structure is tested for in the ordered RA matrix. If the number of embedded absences in the observed site-by-species matrix contains significantly more embedded absences than expected by chance, the distribution is said to be "checkerboard", resulting from several factors including trade-offs in the competitive ability between species, different habitat preferences and other historical factors [24,25,43]. If, on the other hand, there are considerably fewer embedded absences than expected by chance, structure is classed as coherent. Finally, if there is no significant difference between the null and observed matrices, distributions are considered random. A z-test is used to determine whether the distribution significantly positively or negatively deviates from the expected, based on the mean and variance of the null distribution.

Positive coherence suggests that species in general are responding in a similar manner to dominant environmental gradients, as defined by the respective RA axis [18]. For positively coherent distributions, the next step is to assess for species turnover patterns, represented by the number of times a species replaces another species between sites in the ordinated matrix. Positive turnover is evident where the range of one species extends beyond that of another at one end of the gradient and vice versa at the other. On the other hand, negative turnover (or nested subsets) is evident where the range of certain species fall within that of another (i.e. nested within another). Again, these distributions are compared against the 1000 null matrices to assess for deviations from random and significance of this deviation. Based on Presley et al. [19], non-significant turnover can then be assigned as a quasi structure. As per coherence, z tests are used to assess this pattern.

To further explore these distributions, matrices are analysed for boundary clumping patterns, which assesses how clumped the edges of species distributions are [18]. We quantified the degree of boundary clumping with Morista's index (*I*) [44], which can be used as an indicator of dispersion of species in metacommunities [18]. Morista's index is compared between the observed and expected distributions with a Chi^2^ test. Positive turnover can be differentiated into evenly spaced (significantly <1), Gleasonian (non-significant) and Clementsian (significantly >1). On the other hand, nested subsets can be differentiated into hyper dispersed species loss (<1), random species loss (non-significant) and clumped species loss (>1).

#### Boosted regression trees

We assessed the link between environmental variables and both taxonomic richness and the main axis of faunal composition gained from the EMS approach for all stream categories/sizes separately using boosted regression trees (BRT; [45,46]) in the package dismo [47,48]. While we explained the structure of the second RA axis, we did not link it with environmental variables as the first axis represents the best arrangement of species ranges. BRT combines machine learning techniques with traditional statistical approaches (i.e. regression) and thus it can be considered an advanced form of regression. Rather than building complex trees, boosting combines large numbers of simple trees to enhance the predictive ability [47,49]. BRT is suited to identifying predictor variables due to not being limited to simple linear relationships, thus we consider it an ideal tool to explore complex linkages between environmental variables and regional assemblage structure.

We used the ‘gbm.step’ function, which uses stage-wise model selection, and we used the Poisson family loss function and Gaussian loss function to predict richness and the main RA axis, respectively. We used 10-fold cross-validation to select the optimal number of trees. This allows for testing the developing model with held-out data, while using the full set of data at some point in the model building process, and therefore improving the predictive ability of the final model. The bag fraction, which randomly selects the fraction of training data for successive trees, was set to 0.5. We set trees to have five splits and set the learning rate to 0.001, with the exception of the large river richness model, which we set to 0.0001 to ensure 1000 trees were reached.

For the three river section datasets (i.e. headwaters, mid-sized, and large rivers), the raw catchment size value of a site was excluded as a predictor in the analysis. This was because these datasets were defined using catchment size ranges (i.e. 0–20 km^2^, 20–100 km^2^, 100–1000 km^2^), and thus inclusion of catchment size would be somewhat repetitive. Nevertheless, for the model including all river sizes, we included catchment size as a continuous predictor variable. Barring catchment size, models were built using the full set of untransformed physical, chemical and land use data, as BRT are robust to collinear predictor variables and has no need for prior data transformation.

We were also interested in whether spatial variables had a strong influence on faunal compositional patterns. Therefore, we included the two PCoA axes in a second set of BRT models, predicting the primary RA axis. This allowed for examining whether environmental influences on regional faunal composition were simply emerging through regional spatial structuring.

Finally, given the region is divided across the middle by a major catchment boundary separating the Weser River to the North and the Main River to the South (Fig 1), we also tested whether this division influenced the model. To do so, we also ran two more BRT models (one for richness and one for the primary RA axis) on the dataset including all rivers. In this, we incorporated a factor variable indicating whether a site belonged to the northern or southern catchment. However, the inclusion of this factor did not alter the models, with a relative influence on the model of 0.84 and 0.50 (scaled between 0 and 100) for taxonomic richness and RA axis 1, respectively. We therefore will not deal with these results further.

We assessed the relative influence of variables on the models, which is calculated based on how often a variable is selected, and how its selection improves the model, and converted to a percentage. We used the cross-validated percent deviance explained and the cross-validated correlation coefficient between observed and fitted values to examine the overall model performance.

## Results

**Fig 2:**
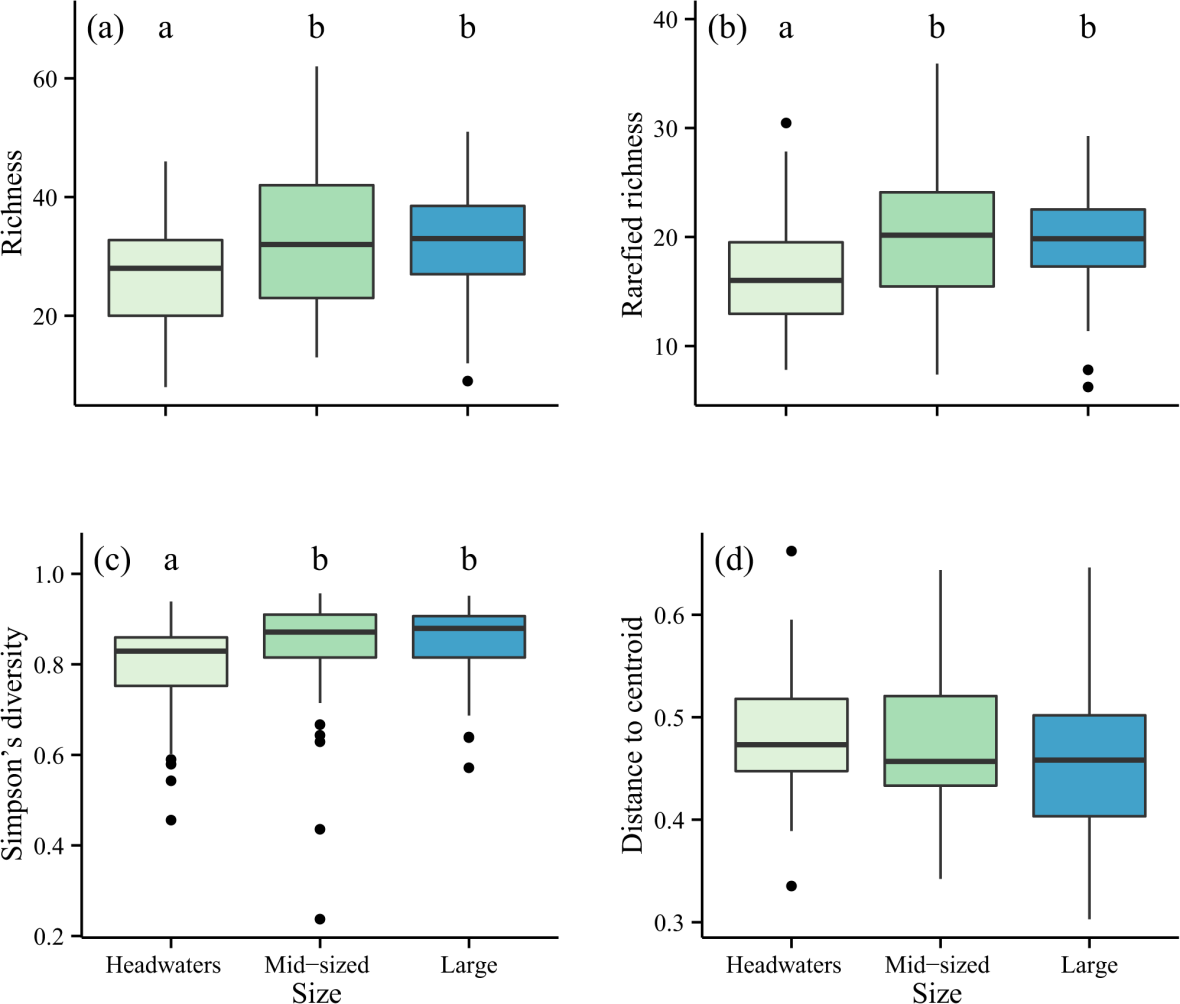
Diversity patterns between river sections. Diversity of benthic macroinvertebrates collected from 168 low-mountain streams and rivers in the German state of Hessen, sampled between 2005 and 2008. (a) Local taxonomic richness, (b) rarefied richness (corrected for 100 organisms), (c) Simpson’s diversity, and (d) beta diversity (beta dispersion, distance to group centroid), between headwaters (light; N = 48), mid-sized streams (medium; N = 69), and large rivers (dark; N = 51). Boxes represent the interquartile range (IQR), and whiskers are the furthest point within 1.5 x IQR above or below the IQR. Values beyond this range are plotted as individual points. Central line represents the median. Local taxonomic richness (*P* < 0.01), rarefied richness (*P* < 0.01), and Simpson’s diversity (*P* < 0.05), but not beta dispersion (*P* > 0.05) differed significantly between the three sections. See text for full results. Different letters on the plot indicate significant differences between stream sections according to Tukey’s HSD test.

Altogether, the dataset of 168 streams and rivers comprised 342 invertebrate taxa. Headwaters had a total richness of 207 taxa, mid-sized streams had 243 taxa and large rivers had 234 taxa. Local taxonomic richness (*F*_2,165_ = 5.63, *P* = 0.004), rarefied richness (*F*_2,165_ = 6.46, P = 0.002) and Simpson’s diversity index (*F*_2,165_ = 3.70, *P* = 0.027), were lower in headwater streams than either of the downstream sections (Fig 2). Despite a trend of decreasing beta diversity from headwaters to large rivers, there was no significant difference between the three sections (*F*_2,165_ = 1.61, *P* = 0.20; Fig 2).

### Regional faunal composition

Regional faunal composition along the primary RA axis (or latent environmental gradient) for all sites combined, headwaters, and mid-sized streams was Clementsian (Table 2). That is, the pattern of faunal composition was positively coherent, consisted of communities with significantly more turnover in space than expected by chance and with clumped boundaries. Large rivers were quasi-Clementsian, resulting from non-significant turnover (Table 2). Similar compositional patterns occurred along the secondary RA axis, with all combined and mid-sized streams having Clementsian distributions, and headwaters and large rivers having quasi-Clementsian distributions. That is, positively coherent, with more than expected but non-significant turnover in space, and clumped boundaries.

**Table 2:** Results of Elements of Metacommunity Structure analysis.

		Coherence					Species turnover					Boundary clumping		
Axis	Stream type	*Abs*	Mean	SD	z	*p*	*Re*	Mean	SD	z	*P*	*MI*	*p*	Best-fit structure
Primary	All	24774	29843.7	769.4	6.59	<0.0001	8419766	1920547.6	780714.1	-8.32	<0.0001	2.89	<0.0001	Clementsian
Primary	Headwaters	3147	3772.4	111.3	5.62	<0.0001	251695	91073.2	29131.8	-5.51	<0.0001	1.75	0.0002	Clementsian
Primary	Mid-sized	6641	8056.4	211.9	6.68	<0.0001	645609	243148.7	89598.9	-4.49	<0.0001	2.002	<0.0001	Clementsian
Primary	Large rivers	5083	5408	165.2	1.97	0.049	247479	153040.5	54087.9	-1.75	0.081	1.794	0.0001	Quasi-Clementsian
Secondary	All	26614	29851.2	767.4	4.22	<0.0001	4389093	1939273.3	793369.7	-3.09	0.002	3.093	<0.0001	Clementsian
Secondary	Headwaters	3405	3771.6	110.6	3.31	0.0009	109176	88730.5	29182.8	-0.7	0.484	1.499	0.004	Quasi-Clementsian
Secondary	Mid-sized	7146	8035.5	203.5	4.37	<0.0001	516855	250088.1	83880.9	-3.18	0.002	3.826	<0.0001	Clementsian
Secondary	Large rivers	4880	5406.7	162.2	3.25	0.0012	174503	156855.1	53394	-0.33	0.741	1.518	0.005	Quasi-Clementsian

Results of Elements of Metacommunity Structure analysis testing for coherence, species range turnover and boundary clumping in 168 low-mountain streams in central Germany categorised into all sites (N = 168), headwaters (N = 48), mid-sized (N = 69) and large rivers (N = 51). *Abs* = number of embedded absences, *Re* = number of replacements, *MI* = Morista’s Index, SD = standard deviation. Both primary and secondary axes of organization are given. Mean and SD values are those calculated from the 1000 generated null matrices.

### Relationship with environmental variables

Richness was best predicted in mid-sized streams with 32% cross-validated (CV) deviance explained (CV correlation between raw and fitted values in model of 0.575 ± 0.086) in the BRT model, but prediction of all streams combined had a similar predictive success (27.8% CV deviance explained; Table 3). Predictive success of headwater communities was much lower (10.6%), and large river richness could not be predicted successfully from the suite of environmental variables (Table 3).

**Fig 3:**
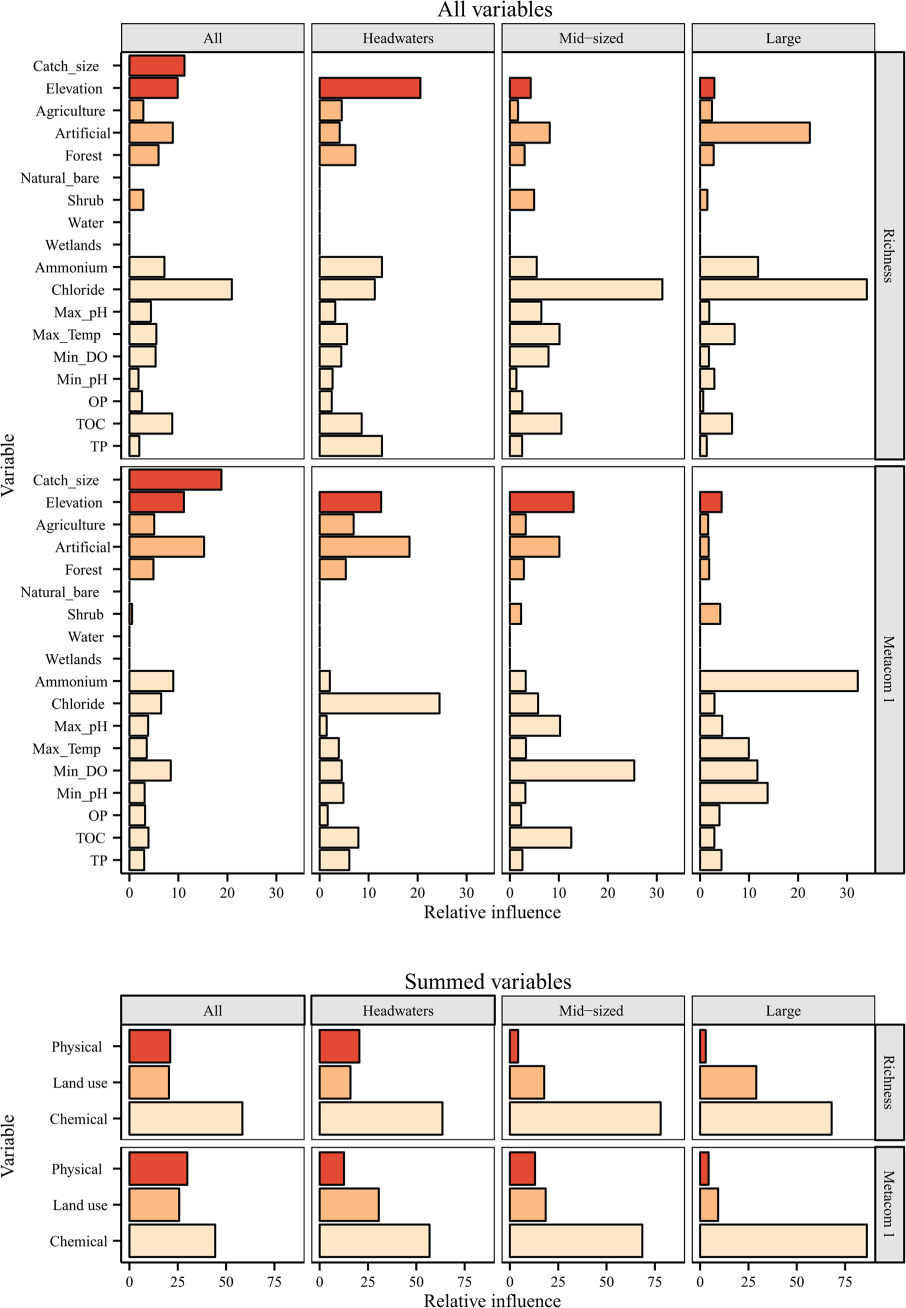
Relative influence of environmental variables in boosted regression tree models. Relative influence of each environmental variable from three categories (physical, land use and chemical) on the boosted regression tree models predicting taxonomic richness and the main axis of regional faunal composition (Metacom 1) in 168 low-mountain streams and rivers in central Germany categorised into all sites (N = 168), headwaters (N = 48), mid-sized (N = 69) and large rivers (N = 51). The top plot shows the relative influence of each individual variable and the bottom the combined influence of each category of variables. Note catchment size was removed from all size-based models (i.e. headwaters, mid-sized and large rivers). See Table 1 for a description of variables and Table 3 for the results of BRT analyses.

Chloride was the most influential variable predicting taxonomic richness for all sites, mid-sized streams and large rivers (Fig 3). Headwater stream richness was strongly influenced by elevation, and the influence of land use increased with stream size, whereas chemical variables varied (Fig 3).

**Table 3:** Results of richness and composition predictions using boosted regression trees.

			Training model				Cross validated		
Dependent	River class	N trees	% deviance explained	Correlation	Mean null deviance	Mean residual deviance	% deviance explained	Correlation (S.E.)	Estimated deviance (S.E.)
Richness	All	2150	60.3	0.83	3.976	1.578	27.8	0.572 (0.041)	2.871 (0.492)
	Headwaters	2600	42.1	0.707	3.256	1.885	10.6	0.472 (0.156)	2.91 (0.426)
	Mid-sized	3400	65.2	0.838	4.638	1.613	32.2	0.575 (0.086)	3.144 (0.555)
	Large rivers	7800	18.5	0.591	2.92	2.38	0.6	0.085 (0.189)	2.902 (0.673)
Regional faunal composition	All	4250	85.4	0.933	0.371	0.054	55.1	0.743 (0.034)	0.166 (0.018)
	Headwaters	7300	79.4	0.897	0.475	0.098	47.5	0.689 (0.108)	0.249 (0.055)
	Mid-sized	7450	85.5	0.931	0.444	0.064	50.4	0.737 (0.066)	0.22 (0.063)
	Large rivers	2700	40.4	0.703	0.313	0.187	13.5	0.321 (0.158)	0.271 (0.069)
Regional faunal composition—with spatial	All	4550	87.2	0.943	0.371	0.048	55.8	0.751 (0.035)	0.164 (0.019)
	Headwaters	5550	76.5	0.883	0.475	0.112	44.3	0.665 (0.12)	0.264 (0.058)
	Mid-sized	6500	84.2	0.926	0.444	0.07	49.2	0.732 (0.066)	0.226 (0.063)
	Large rivers	5700	58.3	0.807	0.313	0.131	17.9	0.471 (0.07)	0.257 (0.072)

Results of the boosted regression tree models predicting taxonomic richness and the primary axis of regional faunal composition in 168 low-mountain streams in central Germany categorised into all sites (N = 168), headwaters (N = 48), mid-sized (N = 69) and large rivers (N = 51).

Faunal composition could be predicted more successfully than richness, with the best predictive explanation for all rivers combined (CV dev. expl. = 55.1%), followed by mid-sized streams (50.4%), headwaters (47.5%) and large rivers (13.5%; Table 3). The strongest variable shaping the axis of faunal composition overall was catchment size, followed by %catchment artificial surfaces and elevation, whereas physicochemical variables had little influence (Fig 3). Headwaters were most strongly influenced by chloride, followed by %catchment artificial surfaces and elevation (Fig 3). Minimum DO had the strongest influence on the mid-sized stream model, followed by TOC, elevation and artificial land cover. Finally, ammonium and other physicochemical variables dominated the large river model (Fig 3). Contrary to richness models, the influence of land use declined with stream size (Fig 3).

**Fig 4:**
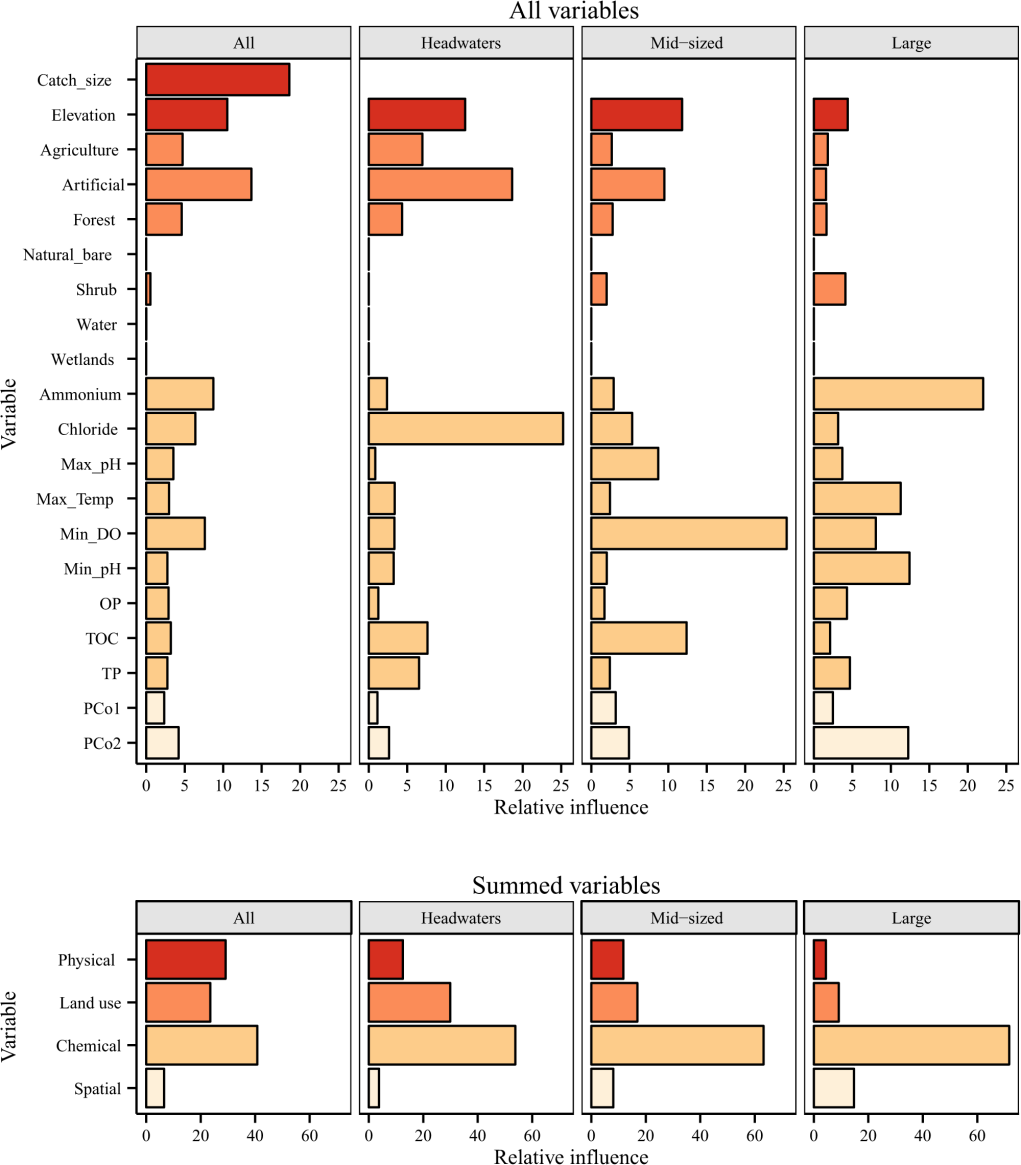
Relative influence of environmental variables in boosted regression tree models including spatial variables. Relative influence of each environmental variable from four categories (physical, land use, chemical, and spatial) on the boosted regression tree models predicting the main axis of regional faunal composition (Metacom 1) in 168 low-mountain streams and rivers in central Germany categorised into all sites (N = 168), headwaters (N = 48), mid-sized (N = 69) and large rivers (N = 51). The top plot shows the relative influence of each individual variable and the bottom the combined influence of each category of variables. Note catchment size was removed from all size-based models (i.e. headwaters, mid-sized and large rivers). See Table 1 for a description of variables and Table 3 for the results of BRT analyses.

Including spatial variables improved the model fit for compositional patterns for large rivers (17.9% CV deviance explained), but not for the other sections (Table 3; Fig 4). Furthermore, relative influences of variables changed little with the inclusion of space, but the importance of spatial variables increased marginally downstream, with combined relative influences of the two PCoA axes of 3.8, 8.1 and 14.7% for headwaters, mid-sized and large rivers, respectively.

## Discussion

### Elements of faunal structure

The best-fit structure for regional compositional patterns of these streams and rivers was Clementsian, with only large rivers having a quasi-Clementsian distribution. Clementsian distributions suggest distinct communities turning over in space, potentially resulting from inter-specific interactions [26], but they could also emerge through other means, including distinct habitat types promoting distinct biotic assemblages. Clementsian structure has been found for many systems and organisms, such as woodlands [50], bats [21], rodents [51], and lake fish communities [52]. A recent study found similar patterns between biologically dissimilar organisms in stream systems, but differences in the idealised structures between different regions: Gleasonian (similar to Clementsian, but with idiosyncratic, rather than grouped turnover) and Clementsian [53]. While there are few previous studies assessing idealised structure of stream metacommunities to compare with, Gleasonian and nested subsets have been found for midges [54], a mix of Clementsian and quasi-Clementsian for fishes [55], and Gleasonian for benthic invertebrates at a small spatial extent [56]. However, it is important to recognise we are observing these patterns at the regional scale. Similar to the primary axis, and despite being orthogonal to the first axis [40], distributions on the secondary RA axis were either Clementsian or quasi-Clementsian. Nevertheless, the fit of the secondary axis was much weaker, which is to be expected given the first axis represents the best arrangement of species ranges [40].

While the EMS approach we have taken is able to identify structural patterns, it does not allow direct identification of the processes underlying these patterns, including dispersal dynamics [57]. However, to examine metacommunity concepts appropriately, sites need to be connected by dispersal, and while this is subjective given our poor understanding of dispersal in stream organisms, the regional extent of our study goes beyond this scale. Nevertheless, the EMS approach allows for examining distributional patterns at biogeographical spatial scales [20–22]. Heino and Alahuta [20] recently examined large-scale regional patterns of beetle fauna and found Clementsian gradients, which they suggest may be common at larger scales due to incorporating or crossing over different species pools. Contrarily, Tonkin et al. [56] found Gleasonian gradients were the most commonly identified structure at small spatial scales for both aquatic and riparian organisms within a single river system. While our approach is at a smaller scale than that of Heino and Alahuta [20], our region crossed catchment boundaries, and thus potentially incorporated different species pools, leading to the observed Clementsian gradients. Nevertheless, including a factor representing whether a site belonged to one of two major drainages, which divide this region, did not contribute to or alter either the richness or metacommunity BRT models.

### Different patterns along the river network

While there was little difference in the predictive ability of environmental variables between headwaters and mid-river sections, environmental control was much weaker in large rivers. Previous work found a clearer SS control in headwaters compared to more highly connected mainstems, where ME increased in importance, reflecting a greater level of isolation of headwaters compared to lower river sections [10]. Consequently, we were interested in whether SS (or simply the predictive ability of environmental variables in this instance) would be stronger in headwaters compared to larger rivers, covering a much larger gradient in both spatial arrangement and river size. While not enough to suggest environmental variables were spatially autocorrelated, the increasing importance of spatial variables from headwaters to large rivers in this study provides some support for a shifting importance from SS to some other paradigm, such as ME or dispersal limitation. Nevertheless, we cannot disentangle the relative role of dispersal in the present study, given the spatial scale being beyond the level where all sites are connected by dispersal. In any case, even where sites are more connected, much of metacommunity research suffers from surrogate measures of dispersal, including spatial arrangement of sites, that do not necessarily represent dispersal accurately [58].

We also asked whether this transition in environmental control would lead to a reduction in beta diversity from headwaters to large rivers. We found that while richness increased downstream, there was only a weak and non-significant trend of decreasing beta diversity. Headwaters often harbour higher beta diversity, including for invertebrates [12] and microbes [11], but also for experimental protozoan systems [59]. It is not only isolation that drives this pattern but other factors may be involved including the fact that headwaters are more abundant [60] and support greater environmental variation [61] than downstream sections. Moreover, these relationships can depend on the life history and ecological specialisation of organisms within river networks [62].

Large rivers had a quasi-Clementsian distribution (resulting from non-significant turnover) and weak coherence, suggesting weaker structuring than the other river sections. This pattern, along with higher alpha and lower beta diversity in these sections, indicates a weakening faunal organisation and environmental control in downstream sections, with communities in these lower sections not reflecting environmental variation as strongly as upstream sites. Nevertheless, multiple metacommunity processes can operate simultaneously [63,64], which can result in weak patterns through opposing forces. Moreover, given the clear role of scale dependence in ecological patterns [65,66], it is important to bear in mind that within a more confined spatial configuration, potentially different distributions may emerge. Scale dependence is not limited to spatial scale, as temporal variability, including dispersal stochasticity [67], can also play a key role in shaping metacommunities [55,56].

### The influence of environmental variables—differences between faunal composition and richness

One of the key differences between the regional faunal compositional pattern and richness models was the finding that catchment size was more important for faunal composition than for alpha diversity. A recent study in Chinese streams found richness to only increase with increasing catchment size in small headwater streams (up to ca. 30 km^2^), levelling off or even declining above this size [68]. It may be inferred from the greater catchment size influence on the metacommunity axis in the present study that a clearer structural shift occurs in faunal composition downstream, than does for richness. In conjunction with the clear differences in the relative influence of key environmental variables, this point answers our fifth question in that environmental predictors differed between richness and faunal composition. There was a much stronger link between environment and faunal composition than there was for richness, and contrary to the patterns in faunal composition, richness was poorly predicted in headwater streams compared to mid-river sections.

As per Brown & Swan [10] we categorised sites into distinct sections of river (but covering a greater gradient in sizes), which potentially masks more complex relationships. Thus, a fruitful area for future research would be to examine linear, rather than categorical, transitions in the competing metacommunity paradigms along river networks. While we were not able to do this in the present study, given the regional extent of our study area, we were able to identify catchment size as the most important predictor of faunal compositional structure along the river network when including all sites.

In general, large rivers could not be predicted well for either regional faunal composition or richness. In response to our fourth question, the importance of chemical constituents on faunal composition increased from headwater to large rivers. However, the influence of environmental variables was somewhat variable for predicting richness patterns. Of the chemical variables, chloride was highlighted as a particularly important driver of richness and faunal composition, but its influence was variable. On the other hand, the influence of elevation and land use decreased in importance downstream. The importance of chloride increased downstream for richness, whereas it was most important for predicting composition in headwaters. Chloride, which can enter rivers from a variety of anthropogenically-derived sources, has been identified as a key stressor in lotic systems and can be highly correlated with other chemical variables such as conductivity [69]. Likewise, ammonium, which was found to be a key factor influencing the large river model, has recently been found to be a key factor influencing large lowland rivers in Germany [70]. These results need to be interpreted with caution however, due to the poor model fit to these large river sites.

## Conclusions

Disentangling spatial and environmental influences on riverine metacommunities has received significant attention in recent years [71–74]. However, important and complementary insights can be gained by examining idealised compositional patterns and their environmental correlates. Our approach, using a powerful statistical technique able to detect complex non-linear relationships (BRT), allowed exploration of key environmental variables shaping regional faunal compositional patterns. Although the only change in best-fit model explaining this regional fauna was from Clementsian to quasi-Clementsian gradients from headwaters to large rivers, key predictors of regional faunal structure differed clearly between sections. The influence of chemical stressors increased from headwaters to large rivers, while the influence of elevation and land use declined.

Heino [15] recently outlined the importance of placing bioassessment programs within a metacommunity framework and recent work has indicated the importance of considering metacommunity dynamics when planning restoration efforts [75,76]. However, studies assessing the influence of environmental stressors on biotic communities have typically focused on detecting changes in richness and other measures of diversity or indices representing community composition. Therefore, it is important to continue examining the alternative ways in which anthropogenic stress can influence lotic metacommunities, such as altering co-occurrence patterns [17] and increasing nestedness [16]. The indication of compartmentalised distributions (Clementsian gradients) suggests that species are turning over in space as groups, rather than individually, which in turn may be reflecting the incorporation of different species pools, given our regional-scale data crossed major catchment boundaries. Our results lend further support to the importance of focusing on a metacommunity or biogeographic framework for both theoretical and applied ecology. Specifically, we found a much stronger link between environmental variables (mostly linked with stressors) and regional faunal composition than with taxonomic richness. In light of this, it is of fundamental importance to continue exploring these patterns within the rapidly developing field of metacommunity ecology, and expand these studies to examine the importance of regional scale [23].
